# Durability of long-lasting insecticidal nets (LLINs) in Ethiopia

**DOI:** 10.1186/s12936-023-04540-3

**Published:** 2023-03-26

**Authors:** Honelgn Nahusenay Hiruy, Seth R. Irish, Semira Abdelmenan, Yonas Wuletaw, Ayele Zewde, Adugna Woyessa, Mebrahtom Haile, Sheleme Chibsa, Lena Lorenz, Alemayehu Worku, Josh Yukich, Yemane Berhane, Joseph Keating

**Affiliations:** 1 Department of Epidemiology and Biostatistics, Addis Continental Institute of Public Health, Addis Ababa, Ethiopia; 2 U.S. President’s Malaria Initiative, Entomology Branch, U.S. Centers for Disease Control and Prevention (CDC), Atlanta, GA USA; 3 Department of Global Health and Policy, Addis Continental Institute of Public Health, Addis Ababa, Ethiopia; 4 Ethiopian Public Health Institute, Addis Ababa, Ethiopia; 5 Ethiopia Federal Ministry of Health, National Malaria Elimination Program, Addis Ababa, Ethiopia; 6 Tulane University School of Public Health and Tropical Medicine, New Orleans, LA USA; 7 U.S. President’s Malaria Initiative, USA Agency for International Development, Addis Ababa, Ethiopia; 8 Department of Disease Control, London School of Hygiene and Tropical Medicine, London, UK; 9 Ifakara Health Institute, Dar-Es-Salaam, Tanzania; 10 College of Medicine and Veterinary Medicine, University of Edinburgh, Edinburgh, Scotland

**Keywords:** Ethiopia, LLIN, Durability, Physical-integrity, Attrition, Survival, Bio-efficacy, Malaria, Deltamethrin, Alpha-cypermethrin

## Abstract

**Background:**

The functional survival time of long-lasting insecticidal nets (LLINs), which varies across different field contexts, is critical for the successful prevention of malaria transmission. However, there is limited data on LLIN durability in field settings in Ethiopia.

**Methods:**

A three-year longitudinal study was conducted to monitor attrition, physical integrity, and bio-efficacy and residual chemical concentration of LLINs in four regions in Ethiopia. World Health Organization (WHO) guidelines were used to determine sample size, measure physical integrity, and calculate attrition rates, and functional survival time. Yearly bio-efficacy testing was done on randomly selected LLINs. An excel tool developed by vector works project was used to calculate the median functional survival time of the LLINs. Predictors of functional survival were identified by fitting binary and multivariate cox proportional hazards model.

**Results:**

A total of 3,396 LLINs were included in the analysis. A total of 3,396 LLINs were included in the analysis. By the end of 36 months, the proportion of LLINs functionally surviving was 12.9% [95% confidence interval (CI) 10.5, 15.6], the rates of attrition due to physical damage and repurposing were 48.8% [95% confidence interval (CI) 45.0, 52.6] and 13.8% [95% confidence interval (CI) 11.6, 14.6], respectively. The estimated median functional survival time was 19 months (95%CI 17, 21). Factors associated with shorter functional survival time include being in a low malaria transmission setting [Adjusted Hazards Ratio (AHR) (95%CI) 1.77 (1.22, 2.55)], rural locations [AHR (95%CI) 1.83 (1.17, 2.84)], and in a room where cooking occurs [AHR (95%CI) 1.28 (1.05, 1.55)]. Bioassay tests revealed that 95.3% (95%CI 86.4, 98.5) of the LLINs met the WHO criteria of bio-efficacy after 24 months of distribution.

**Conclusion:**

The LLIN survival time was shorter than the expected three years due to high attrition rates and rapid loss of physical integrity. National malaria programmes may consider, procuring more durable LLINs, educating communities on how to prevent damage of LLINs, and revising the current three-year LLIN distribution schedule to ensure sufficient protection is provided by LLINs against malaria transmission. While this paper contributes to the understanding of determinants impacting functional survival, further research is needed to understand factors for the rapid attrition rates and loss of physical integrity of LLINs in field settings.

## Background

Malaria prevention and control programmes globally depend on vector control interventions, such as distribution of long-lasting insecticidal nets (LLINs). LLINs are mosquito nets made of material into which insecticide is incorporated or bound around the fibers. They are expected to retain their biological activity for at least 3 years under field conditions [1]. LLINs provide personal protection against malaria by serving as a physical barrier to protect humans from vector contact and by utilizing insecticides to kill vectors. They also reduce transmission and can protect an entire community by mass effect if sustained high functional coverage is attained [2]. However, the protective durability of LLINs has shown significant variation in different field contexts [3, 4]. Durability of LLINs depends on three components: (1) attrition (loss of nets from the household), (2) physical integrity (holes and tears in nets) and (3) insecticidal activity (the amount of residual chemical and its killing effect) [3].

Attrition (complete loss of LLINs) could occur for three different reasons. First, LLINs might be discarded because they are physically damaged and considered non-functional by owners. Second, LLINs might be given away to others. Third, LLINs might be repurposed for unintended uses. These causes of attrition are referred as attrition type one, two and three, respectively [3]. The share of types of attrition varies over time. Immediately after distribution, attrition type 2 (i.e. removal) accounts for the majority of the total attrition [5, 6]. As time goes on, type 1 attrition (i.e. reported physical damage) accounts for an increased amount of the total attrition [7, 8]. Despite claims about the misuse of insecticidal nets [9], the share of type 3 attrition (i.e. use of LLINs for unintended purposes, such as fishing) is reported to be low in African settings [7, 8, 10]. In a 2018 study in central Ethiopia, all cause attrition rate was reported to be 96% within 24 months of follow up [11].

Physical integrity refers to the number and size of holes and tears on the surface of LLIN. It is measured using a composite indicator called proportionate hole index (pHI). Using this index LLINs are classified as being in good, acceptable, or torn condition [3]. The mechanisms by which holes are formed on LLINs were identified to be mechanical (such as sharp materials, and bed edges), thermal, animal damages and seam failure in the order of their contribution [12]. The proportion of LLINs in central Ethiopia that were too torn was 23.1% after 24 months [11].

The interplay between attrition rates and physical integrity determines the functional survival time of LLINs, which varies considerably between countries. Reports have ranged from one year in Ethiopia [11] to two years in Benin [13], three years in Zambia [14], and four years in Uganda [15], and 4.7 years in Zamfara region in Nigeria [4]. The factors that lead to such a variation could be grouped into two broad groups: intrinsic and extrinsic factors [3].

Intrinsic factors refer to manufacturing characteristics of LLINs such as material composition, knitting pattern, quality of finishing, insecticide type and content, additives, and technology used [3]. These factors were found to have statistically significant associations with physical integrity of LLINs and their functional survival time. For example, monofilament yarn polyethylene nets were significantly stronger than the multifilament polyester nets in laboratory experiments by bursting and tension strength [16], and in field settings [5, 8, 17]. Nets with rhomboid knitting patterns (four sided holes) were stronger than hexagonal knitting patterns (six sided holes) [16]. Higher denier values were found to be associated with strong physical integrity [16, 18]. The brand of LLINs has also been reported to be significantly associated with physical integrity, and functional survival time of LLINs [8, 10, 19–21].

Extrinsic factors consist of different aspects of the environment ranging from ecology to the bed type in which the LLIN is used. Ecological factors, such as malaria transmission setting, proximity to water bodies and mosquito breeding sites [11], household factors including housing structure, wealth, socio-economic status of household, knowledge attitude about bed nets [4], net use factors such as type of sleeping space, frequency of wash, and handling practices [11, 13, 14], and user level factors (i.e. number of people who sleep under the net, and age of the users) [8] were found to be significantly associated with durability of LLINs.

While there are a growing number of publications regarding the durability of LLINs in general, the number of studies conducted in Ethiopia are limited. In addition, most of them were retrospective which makes them prone to recall bias [22]. The only prospective study conducted in Ethiopia was done in one geographical area [11]. Since malaria distribution is highly heterogenous, and LLIN functional survival varies across geographical locations [4], there is a need to assess the durability of LLINs in different settings across Ethiopia. This study aims to assess attrition rate, physical integrity, and insecticidal activity of LLINs distributed in the 2015 campaign and identify predictors of LLIN functional survival.

## Methods

### Study setting

**Fig. 1:**
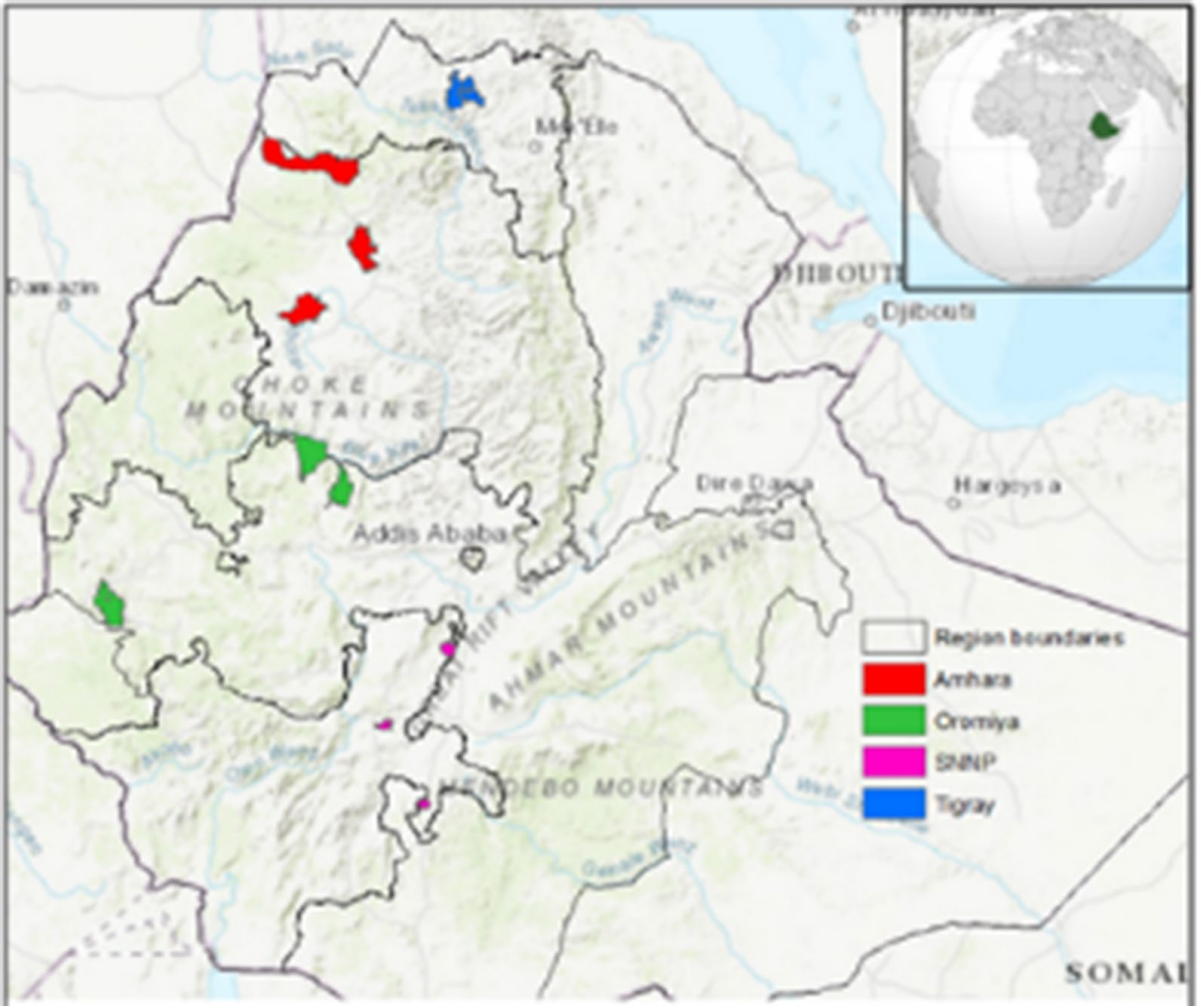
LLIN durability monitoring sites in four regions in Ethiopia, 2015–2018

The study was conducted in 12 districts in four study sites in Ethiopia, that represent different malaria transmission settings (Fig. 1). Ethiopia is divided into 10 regions and two city council administrative units. The regions are further divided into zones, the zones into *woredas* (districts) and the woredas into *kebeles*. The four regions where the study was conducted constitute 86% of the total Ethiopian population [23].

*Anopheles arabiensis* is the predominant vector in Ethiopia, with *Anopheles pharoensis*, *Anopheles coustani*, *Anopheles funestus, Anopheles nili,* and recently *Anopheles stephensi* having a minor role in transmission. *Plasmodium falciparum* accounted for ~ 60% of malaria cases (range 55–69%) and *Plasmodium vivax* 40% (range 31–45%) from 2001 to 2016 [24, 25]. The main malaria prevention and control interventions include LLINs, indoor residual spraying (IRS), early treatment of cases and behavioural change communication. LLINs are distributed through free mass distribution campaigns held every three years for all households in endemic areas below 2000 m altitude [25].

### Study design

This study followed a cohort of LLINs distributed in 2015 over a 3 year period. Single LLINs were the unit of observation and yearly household visits were made to evaluate the presence of LLINs and their physical integrity.

### Sample size

The sample size was calculated following the World Health Organization (WHO) phase III field trial guidelines [3]. The four study sites (regions) were treated as separate survey domains. By assuming a 95 percent confidence interval (CI) and 80 percent power, and attrition rate of 20 percent per year and 50 percent over 3 years, the calculation yielded 460 households for each domain making the total sample size 1840 households. All LLINs in the selected households were included in the study.

Separate sample size was determined for the bioassay and chemical analysis. As per the guideline 40 LLINs were randomly selected from each study site in each survey making the sample size per survey 120 [3].

### Sampling procedure

Details of the sampling procedure are presented in previous publication [26]. In summary, the study treated each of the four study regions as a separate sampling domain and deployed a multistage cluster sampling procedure. First, twelve districts (three from each region) were selected representing to, the extent possible, the three different malaria transmission strata and those distributing campaign nets in the month before the data collection or had plans to distribute nets during the data collection period. Second, a total of 92 clusters (i.e., enumeration areas, EA), 23 from each region, were randomly selected. The number of EAs per district were proportional to the size of the district’s population. Third, 20 households per EA were selected using systematic random sampling procedures, using lists generated by the data collectors in the field. All LLINs received in the selected households from the 2015 distribution campaign were included in the study. They were tagged using a plastic insignia engraved with a unique number, and they were followed annually for three years. The sample LLINs for the bioassay and chemical analysis were randomly selected from households that were not included in the cohort but reside within the same enumeration area.

### Data collection

Baseline data was collected in June 2015, immediately following the national mass LLIN distribution campaign. Follow-up surveys were conducted in June of 2016, 2017 and 2018. Data collection was done using four methods: (1) interviews with heads of households, (2) physical inspections of LLINs, (3) bioassays (4) measurements of the residual insecticide on net samples.

Interviews with heads or any adult member of household’s heads were done using structured questionnaires adopted from WHO guidelines [3]. The questionnaire was used to collect information about the housing characteristics, knowledge and attitude towards LLINs, LLIN handling practices, and reasons for missing LLINs. The same data collection tool, with slight modifications, was used in all follow up surveys.

Inspection of LLINs was done in follow up surveys 12, 24 and 36 months after baseline data collection, following WHO guidelines [3]. The LLINs were inspected outdoors, after draping them over a metal frame. The holes in the LLINs (including tears in the netting and split seams) were measured using a tape measure and their size and location were recorded. The diameter of holes was measured in the longest dimension. Holes with diameter below 0.5 cm were ignored as they were unlikely to allow mosquitos to pass through. In addition, evidence of repairs and types of repairs were recorded. Since all LLINs were brand new, no physical inspection was done at baseline.

Bio-efficacy of LLINs was assessed using the WHO cone test. The cone test involves placing susceptible mosquitoes in contact with net samples for 3 min to see if contact is sufficient to result in a > 95% knockdown one hour after exposure or > 80% mortality 24 h after exposure [3]. The bioassays were conducted with a lab-reared pyrethroid-susceptible strain of *Anopheles arabiensis*, the primary malaria vector in Ethiopia.

Chemical residue analysis was done at baseline and in all three follow up surveys. At baseline, five pieces of netting measuring 30 cm × 30 cm were cut from separate positions, following the WHO sampling scheme. In subsequent surveys, the piece from position 1 was excluded as it was assumed to be tucked under the bed and exposed to excessive abrasion. Net samples were measured to estimate their density (mass of net per unit area), and then samples from the same net were combined for chemical analysis. Chemical content of deltamethrin and alpha-cypermethrin was measured using high-performance liquid chromatography (HPLC), and gas chromatography (GC), respectively.

Field data collection was done by trained data collectors and supervisors using a hand-held tablet with an electronic questionnaire designed using Open Data Kit (ODK) [27]. Data were reviewed and sent to a designated server daily, or as soon as internet connectivity allowed. The data management team downloaded and reviewed data daily. The team provided feedback to data collectors and supervisors in the field as needed. Bioassays were done at the Adama insectary while chemical analysis was done at the Adami Tulu Pesticides Processing S.C. laboratory.

### Measurements

*Malaria transmission setting:* Using Annual Parasite Incidence (API), districts were classified as low (API < 5/1000), moderate (API 5–100/1000) and high (API >  = 100/1000) malaria transmission settings.

*Perception towards net care and repair* was measured by asking a series of eight Likert-scale statements, with response captured as—2 “strongly disagree”, − 1 “disagree”, 0 “neutral”, 1 “agree”, or 2 “strongly agree”. After calculating the average score, respondents were categorized as having negative (< 0), positive (score between 0.01 and 1.0), or very positive (score between 1.01 and 2.0) perceptions toward net care and repair. Details of the method are described in a previous publication [26].

*Economic wealth status* of households was measured based on a composite measure of wealth index based on household assets and housing conditions [28], then categorized into quintiles.

Box 1 Shows the durability indicators measured by this study. All indicators were measured using WHO definitions.

**Table:** 

Box 1: durability monitoring indicator calculation	
$${\text{All cause attrition rate at time T}}_{{\text{i}}} \, = \,\frac{{{\text{Total LLINs under follow up reported as missing from households at}}\,T_{i} }}{{{\text{Total LLINs enrolled for follow up at time}}\,T_{0} }}\,{\text{X}}\,100$$	
$${\text{Attrition rate}} - {1}\left( {\text{Physical damage}} \right){\text{ at time T}}_{{\text{i}}} \, = \,\frac{{{\text{Total LLINs under follow up reported as thrown out due to wear and tear at time}}\,T_{i} }}{{{\text{Total LLINs enrolled for follow up at time}}\,T_{0} }}\,{\text{X}}\,100$$	
$${\text{Attrition rate}} - {2}\left( {{\text{Removal}}} \right){\text{ at time T}}_{{\text{i}}} = \frac{{{\text{Total LLINs under follow up reported as given away}},{\text{ stolen}},{\text{ sold or used in another location at time}}\,T_{i} }}{{{\text{Total LLINs enrolled for follow up at time}}\,T_{0} }}\,{\text{X}}\,100$$	
$${\text{Attrition rate}} - {3}\left( {{\text{Re}} - {\text{purposed}}} \right){\text{ at time T}}_{{\text{i}}} = \frac{{{\text{Total LLINs under follow up reported as being used for another purpose at time}}\,T_{i} }}{{{\text{Total LLINs enrolled for follow up at}}\,T_{0} }}\,{\text{X}}\,100$$	
*Size 1 hole* = *Hole with diameter 0.5–2.0 cm*	
*Size 2 hole* = *Hole with diameter 2–10 cm*	
*Size 3 hole* = *Hole with diameter 10–25 cm*	
*Size 4 hole* = *Hole with diameter* > *25 cm*	
*pHI* = *# size 1 holes* + *(# size 2 holes* × *23)* + *(# size 3 holes* × *196)* + *(# size 4 holes* × *576)*	
Good = total hole surface area < 0.01m^2^ or pHI < 64	
Acceptable = total hole surface area < = 0.1 m^2^ or pHI 64–642	
Torn = total hole surface area > 0.1m^2^ or pHI > 642	
$${\text{Proportion surviving in functional condition }} = \frac{{{\text{LLINs found in households with no hole}} + {\text{in good}} + {\text{acceptable condition }}{-}{\text{unknown status}}}}{{{\text{Total LLINs enrolled for follow up at}}\,T_{0} {-}{\text{Given away }}{-}{\text{unknown status}}}}\,{\text{X}}\,100$$	
Median Survival time = ${\text{t}}_{{1}} + \,\frac{{\left( {{\text{t}}_{{2}} - {\text{t}}_{{1}} } \right) - \left( {{\text{P}}_{{1}} - {5}0} \right)}}{{\left( {{\text{P}}_{{1}} - {\text{P}}_{{2}} } \right)}}$ *t*_*1*_*: first time point, t*_*2*_*: Second time point, P*_*1*_*: functional survival at t*_*1*_*, P*_*2*_*: functional survival at t*_*2*_	
Bio-efficacy = A candidate LLIN is considered to meet the criteria for efficacy for testing in phase III studies if, after 3 years, at least 80% of sampled nets are effective in WHO cone tests (≥ 95% knockdown or ≥ 80% mortality)

### Data analysis

Data analysis involved the calculation of durability indicators and identification of predictors for LLIN functional survival. The analysis was done using Stata version 15 [29]***.*** In doing so the “*svvyset*” command was used to account for the complex survey data. Population weights were applied to account for unequal probability of selection across the districts, and predictors of functional survival by fitting Cox proportional regression model. The outcome event was defined using two criteria. LLINs not found in their respective households due to attrition type 1 (physical damage) or attrition type 3 (repurposed) and those that were found in torn physical condition were defined as developing the outcome event. On the other hand, LLINs found in their respective households with no holes, or in good or acceptable physical condition, and missing LLINs because of attrition type 2 (given away) were defined as not developing the outcome of event. Survival time was measured in months. It was calculated as the duration between starting of the follow up time and the time in which the event happened. For those LLINs that were physically inspected, the time of survey was taken as time of event. For LLINs missing from households, respondents were asked to estimate the time they disposed of or gave away their LLINs.

The model construction began by testing for presence of association between literature based on pre-identified factors, and the outcome variable using bivariate Cox proportional regression. Then variables having a P-value of < 0.25 was included in the multivariate regression analysis. This P-value cut off point has been used in other studies [11]. After fitting the final model, the model was tested for the fulfillment of the proportional hazards’ assumption using test of nonzero slope in a generalized linear regression of the scaled Shonefeld residuals, using *“estat phtest, detail”* command in Stata. The global test revealed no evidence of violation of the assumption. However, the test for each factor revealed that one factor (i.e., study site) violates the proportional-hazards assumption and it was excluded from the final model.

### Ethical considerations

The study protocol was approved by the Institutional Review Board (IRB) at Addis Continental Institute of Public Health (ACIPH), which is a nationally registered board. Upon approval, permission letters were obtained from the four-regional health bureaux. Informed consent was obtained from each study respondent. Personal identifiers were kept in strict confidentiality and were used only for follow-up purposes. LLINs sampled for bioassay and chemical analysis were removed from their respective households after securing consent, and they were replaced by new LLINs.

## Results

### Baseline characteristics of LLINs and follow up completion

A total of 3,396 LLINs were included in the analysis. One third (33.9%) of them were PermaNet 2.0^®^ while the rest were MAGNet^®^. Most of the LLINs were obtained from rural areas (93.3%), moderate malaria transmission settings (76.8%) and households whose head had no formal education (51.9%). A little more than half (53.5%) of the LLINs were owned by household heads that have positive perception towards net care and repair. Half (50.3%) of LLINs were owned by households that never cook in their sleeping rooms (See Table 1).

**Table 1:** Baseline characteristics of LLINs enrolled, Ethiopia, 2015–2018

Variables (n = 3396)	Unweighted frequency	Weighted proportion	(95% CI)
Study site			
Tigray	886	24.7	(21.3, 28.4)
Amhara	723	22.5	(18.5, 27.0)
Oromia	1043	33.4	(29.4, 37.7)
SNNPR	744	19.4	(16.0, 23.4)
Malaria transmission setting			
Low (API < 5/1000)	777	19.7	(12.7, 29.3)
Moderate (API 5–100/1000)	2476	76.8	(66.8, 29.3)
High (API > = 100/1000)	143	3.4	(1.6, 7.3)
Residence			
Urban	261	6.7	(3.4, 13.0)
Rural	3135	93.3	(87.0, 96.6)
Household head gender			
Male	2859	85.3	(83.1, 87.3)
Female	513	14.7	(12.7, 16.9)
Household head mean age (SD)	44.75	13.67	
Educational status of head of household			
No formal education	1777	51.9	(47.6, 56.1)
Primary (grade 1–6)	935	28.0	(25.1, 31.1)
Secondary (grade 7–8)	282	8.4	(6.8, 10.3)
High School (grade 9–10)	209	6.4	(5.0, 8.0)
Above high school	176	5.4	(3.5, 8.2)
Exposure to information on net care and repair			
No	2518	75.0	(71.3, 78.3)
Yes	878	25.0	(21.7, 28.7)
Knowledge about net care and repair			
Not Adequate	2226	65.1	(60.5, 69.6)
Adequate	1159	34.9	(30.4, 39.5)
Perception towards net care and repair			
Negative	800	24.0	(21.2, 27.0)
Positive	1800	53.5	(49.9, 57.1)
Very positive	785	22.5	(19.5, 25.9)
Mean family size (SD)	5.6	2.1	
Wealth index			
Lowest	690	20.1	(16.1, 24.8)
Second	639	18.9	(15.9, 22.2)
Middle	688	20.4	(17.3, 23.8)
Fourth	770	23.1	(19.6, 27.0)
Highest	608	17.6	(13.0, 23.3)
House infested with rodents			
No	675	20.1	(17.0, 23.8)
Yes	2721	79.9	(76.2, 83.0)
Cooking in sleeping rooms			
Always	983	27.2	(23.6, 31.2)
Most of the time	278	8.6	(6.9, 10.6)
Sometimes	445	13.8	(11.6, 16.4)
Never	1663	50.3	(46.8, 53.9)
Brand			
MAGNet®	2280	66.1	(57.9, 73.5)
PermaNet 2.0®	1116	33.9	(26.5, 42.1)
Sleeping place LLIN used over			
Bed frame (finished)	240	7.0	(5.4, 9.1)
Bed frame (sticks)	637	18.2	(14.5, 22.5)
Foam mattress	43	1.3	(0.5, 3.2)
Reed mattress	25	0.7	(0.4, 1.2)
Grass mattress	341	9.7	(6.7, 13.8)
Floor with no mattress	224	6.8	(5.1, 8.9)
LLIN never used	1886	56.4	(51.4, 61.2)
Number of nights net was used last week			
Every night (7 nights)	1019	29.5	(25.2, 34.1)
Most nights (5–6 nights)	77	2.2	(1.5, 3.0)
Some nights (1–4 nights)	249	6.6	(3.8, 11.3)
Not used last week	140	4.4	(3.4, 5.6)
Net never used at all	1904	57.4	(52.3, 62.3)

By the end of the third year, a definite outcome was obtained for 3155 LLINs, making the follow up completion 93.0% (95%CI 91.3, 94.4). The most common reason for loss to follow-up was households moving away, not being available during data collection, and respondents being unable to recall what happened to LLINs, which were no longer available in their house. The baseline characteristics of LLINs that were lost to follow up were compared against those that completed the follow up and no statistically significant difference was found.

### Attrition

**Fig. 2:**
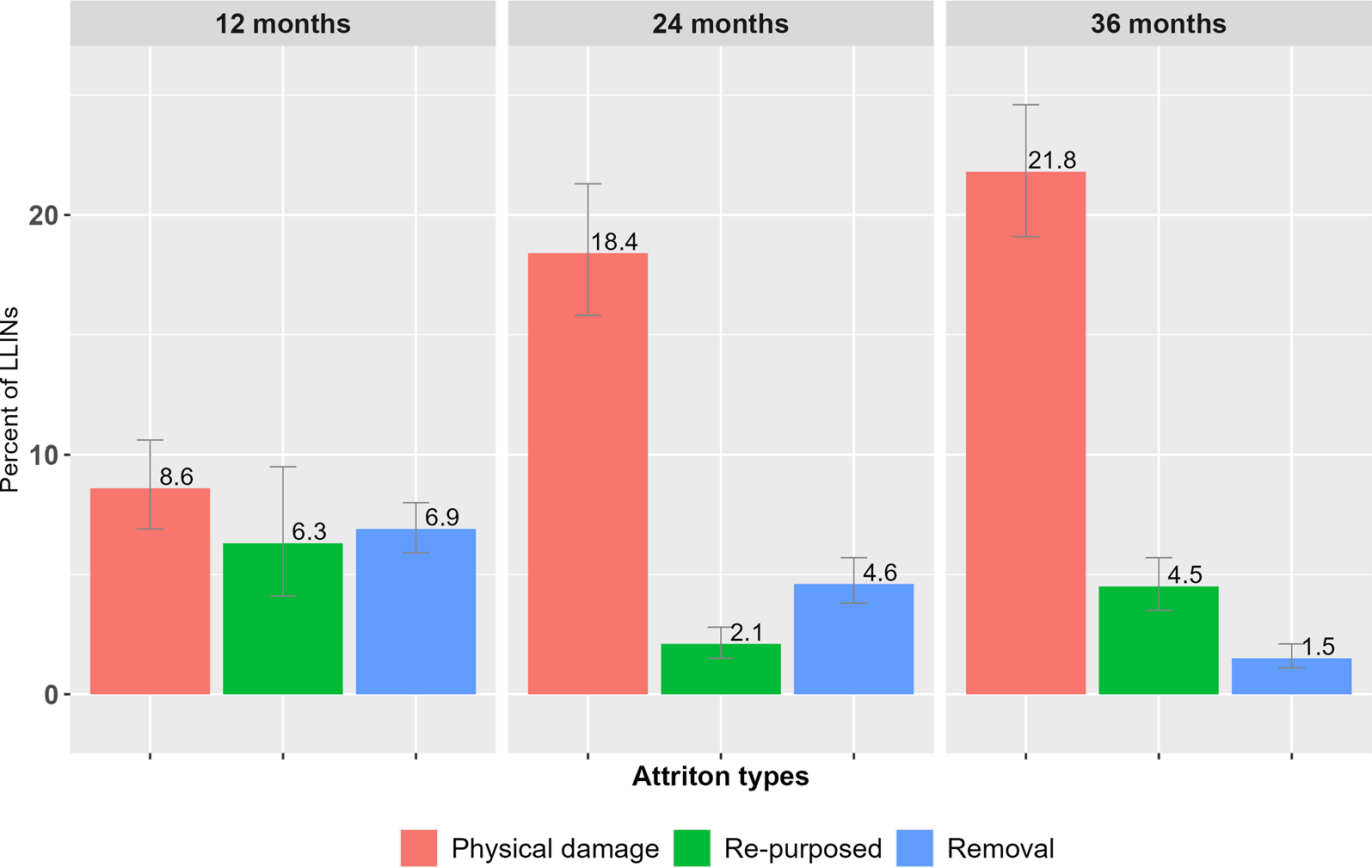
Attrition of LLINs in four study regions in Ethiopia, 2015–2018

Out of the 3396 LLINs tagged for follow up, 2596 (77.1%) of LLINs were lost by the third year due to physical damage (48.8%), removal (i.e., giving away for others) (13.0%), and repurposing (12.8%). In all rounds of surveys, physical damage was reported to be the leading cause, followed by removal and repurposing (See Table 2 and Fig. 2).

**Table 2:** Attrition rates of LLINs in four study regions in Ethiopia, 2015–2018

(n = 3,396)	12 months			24 months			36 months			Total		
	n	%	(95CI)	n	%	(95CI)	n	%	(95CI)	n	%	**(**95CI**)**
Attrition rate 1 (Physical damage)	300	8.6	(6.9, 10.6)	645	18.4	(15.8, 21.3)	716	21.8	(19.1, 24.6)	1661	48.8	(45.0, 52.6)
Attrition rate 2 (Removal)	225	6.9	(5.9, 8.0)	155	4.6	(3.8, 5.7)	54	1.5	(1.1, 2.1)	434	13.0	(11.6, 14.6)
Attrition rate 3 (Re-purposed)	194	6.3	(4.1, 9.5)	68	2.1	(1.5, 2.8)	147	4.5	(3.5, 5.7)	409	12.8	(10.1, 16.0)
Unknown	24	0.8	(0.4, 1.6)	41	1.0	(0.6, 1.5)	27	0.7	(0.4, 1.1)	92	2.5	(1.8, 3.3)
Total	743	22.5	(18.5, 27.1)	909	26.1	(23.2, 29.3)	944	28.4	(25.5, 31.5)	2,596	77.1	(73.5, 80.3)

*n* unweighted counts, % weighted rates

### Physical integrity

**Fig. 3:**
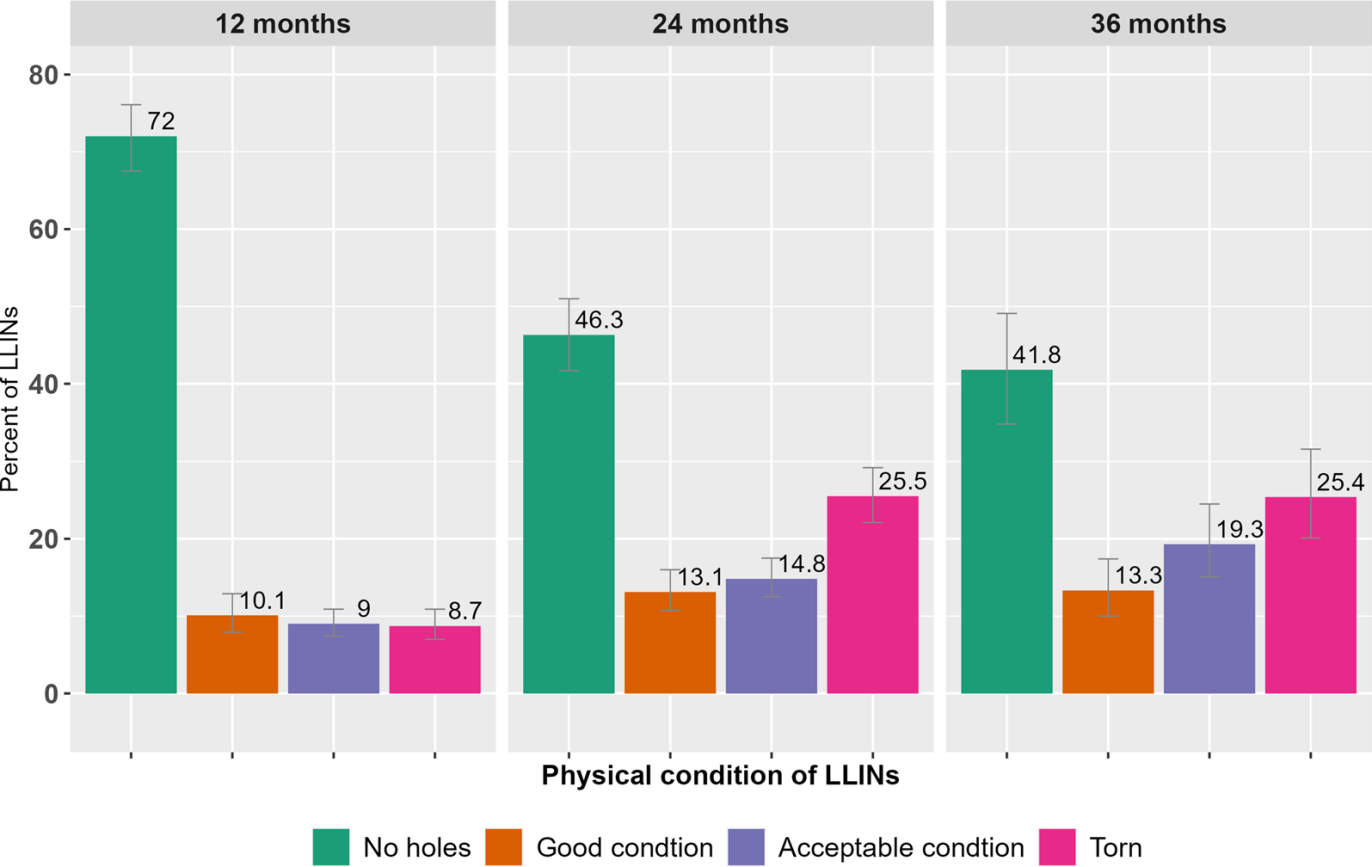
Physical integrity of LLINs in four study regions in Ethiopia, 2015–18

Out of the 3396 LLINs recruited for follow up, 2,440, 1,476 and 536 were available for inspection at 12, 24 and 36 months, respectively. Out of these LLINs, the proportion found in torn condition were 8.7% at 12 months, 25.5% at 24 months, and 25.4% at 36 months of follow up after distribution. (See Table 3 and Fig. 3).

**Table 3:** Physical integrity of LLINs in four study regions in Ethiopia, 2015–18

Physical condition of LLINs	12 months			24 months			36 months		
	n	%	(95%CI)	n	%	(95%CI)	n	%	(95%CI)
LLINs observed	2440			1476			536		
No holes	1752	72.0	(67.5, 76.1)	690	46.3	(41.7, 51.0)	226	41.8	(34.8, 49.1)
Good condition (pHI < 64)	242	10.1	(7.9, 12.9)	181	13.1	(10.7, 16.0)	68	13.3	(10.0, 17.4)
Acceptable condition (pHI: 64–642)	227	9.0	(7.4, 10.9)	221	14.8	(12.5, 17.5)	95	19.3	(15.1, 24.5)
Torn (pHI > 642)	215	8.7	(7.0, 10.9)	381	25.5	(22.1, 29.2)	146	25.4	(20.1, 31.6)
Unknown	4	0.1	(0.0, 0.5)	3	0.3	(0.1, 0.8)	1	0.1	(0.0, 1.0)

### Functional survival

**Fig. 4:**
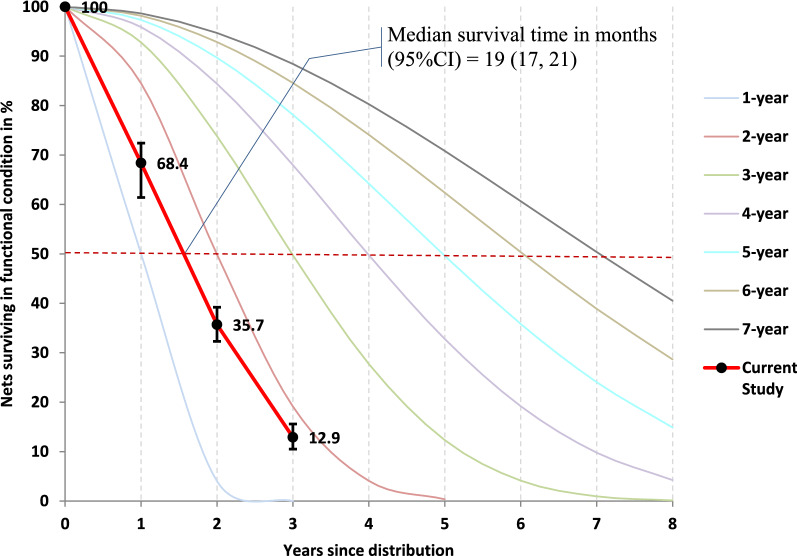
Estimated net survival in functional condition with 95% confidence intervals plotted against hypothetical survival curves in fours study sites in Ethiopia, 2015–2018

After excluding LLINs that were given away, the proportion of LLINs surviving in a functional status (i.e., with no holes, or in acceptable or good condition) were 68.4% (2,221) in the first year of follow up. These percentages decreased to 35.7% (1,092) in the second year and 12.9% (389) by the third year. The median functional survival time, time by which 50% of LLINs were in serviceable condition, was 19 months (95%CI = 17, 21). The observed survival curve plotted against the loss prediction curves was between one- and two-years serviceable models (See Fig. 4).

### Predictors of functional survival

Multivariate proportional Cox regression model identified important independent predictors of functional survival time. Factors that lead to shorter functional survival time included: being in a low or moderate malaria transmission setting, rural residency, increase in family size, cooking inside sleeping room, and being the lowest, second or fourth wealth quintile.

LLINs in low malaria transmission settings were more likely to have shorter functional survival time [AHR (95%CI) 1.77 (1.22, 2.55)] compared to those in high transmission settings. LLINs owned by rural residents had a shorter [AHR (95%CI) 1.83 (1.17, 2.84)] functional survival time compared to LLINs owned by urban dwellers. As family size increased, LLINs tended to have shorter functional survival time [AHR (95%CI) 1.10 (1.05, 1.14)]. LLINs owned by households in the lowest, second, and fourth wealth quintiles had shorter functional survival time compared to those in highest wealth quintile (See Table 4 for AHR and 95%CI). LLINs owned by households that always cooked in their sleeping rooms had a shorter survival time [AHR (95%CI) 1.23 (1.01, 1.50)] compared to those that never cooked in the sleeping rooms (See Table 4).

**Table 4:** Predictors of failure to functional survival of LLINs over 36 months of follow up in four study regions in Ethiopia, 2015–2018

Variables	CHR	(95% CI)	p value	AHR^a^	(95%CI)	p value
Residence						
Urban	Ref.			Ref.		
Rural	3.79	(2.99, 4.80)	0.00	**1.83**	**(1.17, 2.84)**	0.01*
Malaria transmission setting						
Low (API < 5/1000)	1.36	(1.08, 1.73)	0.01	**1.77**	**(1.22, 2.55)**	0.00*
Moderate (API 5—100/1000)	1.22	(0.97, 1.53)	0.09	1.40	(1.00, 1.96)	0.05
High (API > = 100/1000)	Ref.			Ref.		
Household head gender						
Male	Ref.			Ref.		
Female	0.70	(0.62, 0.80)	0.00	0.98	(0.78, 1.23)	0.84
Household head mean age	0.99	(0.99, 1.00)	0.00	1.00	(0.99, 1.01)	0.72
Educational status of head of household						
No formal education	1.75	(1.39, 2.19)	0.00	1.23	(0.86, 1.77)	0.26
Primary (grade 1–6)	1.69	(1.34, 2.13)	0.00	1.21	(0.85, 1.72)	0.30
Secondary (grade 7–8)	1.75	(1.35, 2.28)	0.00	1.41	(0.93, 2.14)	0.11
High School (grade 9–10)	1.54	(1.17, 2.05)	0.00	1.34	(0.86, 2.08)	0.20
Above high school	Ref.			Ref.		
Exposure to information on net care and repair						
No	Ref.			Ref.		
Yes	0.88	(0.80, 0.97)	0.01	0.90	(0.75, 1.07)	0.23
Knowledge about net care and repair						
Not Adequate	Ref.					
Adequate	1.07	(0.98, 1.16)	0.15	1.04	(0.89, 1.22)	0.64
Perception towards net care and repair						
Negative	1.07	(0.94, 1.20)	0.30	0.92	(0.74, 1.15)	0.45
Positive	1.00	(0.90, 1.12)	0.93	0.96	(0.80, 1.15)	0.66
Very positive	Ref.			Ref.		
Mean family size (SD)	1.06	(1.04, 1.08)	0.00	**1.10**	**(1.05, 1.14)**	0.00*
Wealth index						
Lowest	2.35	(2.03, 2.72)	0.00	**1.50**	**(1.08, 2.08)**	0.01*
Second	2.33	(2.01, 2.71)	0.00	**1.70**	**(1.26, 2.08)**	0.00*
Middle	1.76	(1.51, 2.05)	0.00	1.15	(0.86, 1.55)	0.35
Fourth	1.64	(1.42, 1.90)	0.00	**1.34**	**(1.03, 1.75)**	0.03*
Highest	Ref.			Ref.		
House infested with rodents						
No	Ref.			Ref.		
Yes	1.26	(1.13, 1.41)	0.00	1.11	(0.92, 1.35)	0.29
Cook in sleeping rooms						
Always	1.06	(0.97, 1.17)	0.21	**1.23**	**(1.01, 1.50)**	0.04*
Mostly	0.95	(0.81, 1.12)	0.53	1.04	(0.78, 1.39)	0.78
Sometimes	0.93	(0.81, 1.06)	0.27	1.02	(0.79, 1.33)	0.88
Never	Ref.			Ref.		
Sleeping place LLIN used over						
Bed frame (finished)	Ref.			Ref.		
Bed frame (sticks)	1.95	(1.60, 2.37)	0.00	1.03	(0.73, 1.44)	0.87
Foam mattress	0.91	(0.57, 1.44)	0.68	0.67	(0.30, 1.52)	0.34
Reed mattress	2.84	(1.80, 4.46)	0.00	1.61	(0.73, 3.55)	0.24
Grass mattress	1.91	(1.54, 2.35)	0.00	1.20	(0.83, 1.72)	0.33
Floor with no mattress	2.06	(1.64, 2.60)	0.00	1.09	(0.71, 1.67)	0.69
Never used	1.26	(1.05, 1.51)	0.01	1.36	(0.82, 2.25)	0.23
Number of nights net was used last week						
Every night (7 nights)	Ref.			Ref.		
Most nights (5–6 nights)	1.27	(0.97, 1.67)	0.09	1.25	(0.81, 1.92)	0.31
Some nights (1–4 nights)	0.92	(0.78, 1.09)	0.33	1.10	(0.84, 1.44)	0.51
Not used last week	0.70	(0.56, 0.88)	0.00	0.68	(0.43, 1.08)	0.10
Net never used at all	0.71	(0.65, 0.78)	0.00	0.65	(0.39, 1.07)	0.09
Place net was dried						
On a cloth line	Ref.			Ref.		
On ground	0.96	(0.81, 1.13)	0.63	1.11	(0.92, 1.34)	0.26
On bush, or fence	1.25	(1.03, 1.52)	0.02	1.09	(0.88, 1.35)	0.43

*CHR* crude hazards ratio, *AHR* adjusted hazards ratio

^a^Also adjusted for brand

^*^p-value < 0.05

### Bioassay results

As per the WHO criteria, LLINs were considered effective, if they resulted in > 95% mosquito knockdown in 1 h or > 80% mortality in 24 h after the exposure. Accordingly, 95.3% (95%CI: 86.4, 98.5) of the LLINs met the criteria of effectiveness 24 months after distribution but only 19.0% (95%CI: 12.6, 27.7) of the LLINs at 36 months (See Table 5).

**Table 5:** Proportion of long-lasting insecticidal nets meeting WHO pesticide evaluation scheme effectivity criteria (1 h knockdown ≥ 95 or 24 h mortality ≥ 80) in Ethiopia, 2015–18

Variable	12 months	24 months	36 months
Total	n = 97	n = 64	n = 105
Proportion and 95 CI of LLINs meeting WHO pesticide evaluation scheme effectivity criteria (1 h knockdown ≥ 95 or 24 h mortality ≥ 80)	100 (na)	95.3 (86.4, 98.5)	19.0 (12.6, 27.7)

### Residual chemical analysis results

Table 6 presents the mean, standard deviation, 95% confidence interval and percentage of residual concentration of alpha-cypermethrin and deltamethrin of MAGNet and PermaNet 2.0 LLINs, respectively. At baseline, the mean concentration of alpha-cypermethrin was 4.64 g/kg with standard deviation of 0.58. By the end of the study (36 months after distribution) the mean concentration was 3.39 g/kg, which is equivalent to 73.33% of the baseline concentration.

**Table 6:** Alpha-cypermethrin content of MAGNet^®^ and deltamethrin content of PermaNet 2.0^®^ LLIN after 12, 24 and 36 months

	Baseline	12 months	24 months	36 months
Alpha-cypermethrin content of MAGNet® LLIN	n = 22	n = 62	n = 47	n = 58
Mean concentration of A.I.^a^ in g/kg	4.64	3.55	3.84	3.39
(Std. Dev.)	(0.58)	(1.12)	(0.95)	(1.43)
(95% CI)	(4.40, 4.88)	(3.27, 3.83)	(3.57, 4.11)	(3.03, 3.77)
Percentage of residual A.I. from baseline	NA	76.64%	82.87%	73.33%
Deltamethrin content of PermaNet 2.0^®^	n = 8	n = 24	n = 14	n = 27
Mean concentration of A.I.^a^ in g/kg	1.91	0.78	0.45	0.47
(Std. Dev.)	(0.24)	(0.36)	(0.38)	(0.47)
(95% CI)	(1.73, 2.06)	(0.63, 0.92)	(0.25, 0.65)	(0.31, 0.62)
Percentage of residual A.I. from baseline	NA	41.07%	23.86%	24.64%

^a^Active ingredient

The mean deltamethrin concentration of PermaNet 2.0 at baseline was 1.91 g/kg (95%CI: 1.73, 2.06). After 24 and 36 months, the mean chemical concentrations were 0.45 g/kg and 0.47 g/kg, resulting in 23.86% and 24.64% of the baseline concentration, respectively (see Table 6).

## Discussion

This study showed that LLINs did not last the recommended three years in the field setting. High levels of attrition (type 1 and 2) combined with rapid deterioration of physical integrity led to shorter functional survival time. Factors that led to shorter functional survival time included being in a low malaria transmission setting, rural residency, large family size, cooking inside sleeping rooms, and lower wealth status. Furthermore, the vast majority LLINs met the criteria of acceptable bio-efficacy up to the end of the second year.

Unlike previous studies, which were retrospective and cross sectional [22], or limited to one geographic setting [11], this study employed a prospective design in different geographic and malaria transmission settings. This study also followed WHO guidelines for durability monitoring of LLINs in the field setting [3]. While these are the strengths of this study, it also has limitations that are worth discussing.

Because of the prospective nature of the study design, households might have tended to keep their LLINs longer than they normally would have, which might have led to an over estimation of the functional survival time. The classification of attrition types was based on the reporting of the owners, which might be prone to recall and social desirability biases. In addition, users might have their own judgments for determining LLINs as “not useful anymore” and discard them. Due to the violation of the proportional hazard assumption, study site could not be included in the final regression model, and this limits the study from assessing the impact of site on functional survival time of LLINs. In the context of these limitations the study come up with important findings that are discussed below.

The estimated median functional survival time was only 19 months (95%CI 17, 21), which was shorter than the expected 36 months [3, 30]. Another study conducted in central Ethiopia reported 12 months of median survival time [11]. This shorter survival time might lead to a reduction in the protection of the community against malaria, especially in the second and third years after LLIN distribution campaigns.

By the end of the third year 48.8% of the LLINs were lost from because of attrition rate 1 (damage) and another 12.8% of LLINs were lost due to attrition rate 3 (repurposing). These two types of attritions have greatly contributed for the shorter functional survival time of LLINs. The fact that most of LLINs were reported to be discarded due to damage might be a proxy indicator for discarding of LLINs even with minor damage, as identified by other studies in Ethiopia in which LLINs with some holes were considered to be too torn [31]. The study also identified that 13.8% of the LLINs were removed (given away) from the house. These LLINs might be in use in other households.

The second contributor for shorter functional survival time was the rapid deterioration of physical integrity of LLINs. A quarter of the available LLINs were in torn condition by the second and third year. This proportion is comparable to studies done in Ethiopia 11], and Zambia [14]. While this study did not assess the cause of each hole on each LLIN, it asked respondents how holes were formed in their LLINs. Accordingly, the common causes reported were mechanical causes (such as sharp objects, and corners of beds) that accounted for 31.49%, and rodents, which contributed 43.61%.

This study has identified important factors that affect the functional survival time of LLINs. LLINs in low malaria transmission settings tended to have shorter functional survival time. LLINs in these settings might be less valued by owners and prematurely disposed. LLINs in rural areas were also found to have shorter functional survival time. This could be due to the difference in living conditions, and household behaviours. Similar findings have been reported in Nigeria [4].

As family size increased, LLINs tended to have shorter functional survival time. Increases in family size might increase the number of individuals sleeping under a net, which has been found to be a risk factor for loss of physical integrity [8].

LLINs owned by households in the lower wealth quantile tended to have shorter functional survival time. This could be due to difference in living conditions. LLINs owned by households that cooked inside their sleeping rooms had a shorter survival time. This could be due to damage of LLINs by fire. Other studies have reported higher risk of losing physical integrity among LLNs owned by households in which kitchens and sleeping spaces are located in the same room [8, 13].

In this study, LLINs retained their bio-efficacy (at least 80% of the sampled LLINs were effective in a WHO cone test) up to 24 months, which was in line with other studies in Ethiopia [11].

The average chemical content of alpha-cypermethrin and deltamethrin at baseline was with the range of WHO specification of 5.8 g/kg ± 25% [32] and 1.8 g/kg ± 25% [32, 33], respectively. However, the chemical content of the 12, 24, and 36 months is below the WHO specification. Unexpectedly, the 24 month chemical concentration was slightly higher than the 12 month value. This could be due to the differences in the sampled nets, and how they were handled in their respective households. Detailed analysis on such differences could not be done, as data on household characteristics and LLIN handling practices was not collected for the nets sampled for the chemical and bioassay analyses. Farther more this could be because the LLNs that were used more frequently might have been discarded already, and the remaining ones are either in better condition or have been handled more carefully prior to sampling them for the chemical analysis.

## Conclusions and recommendation

In general, this study identified that LLINs are lasting shorter than the expected three years. This was due to a high type 1 attrition rate and loss of physical integrity of remaining LLINs. The National Malaria Programme might need to consider procuring more durable LLINs, educate the community on how to prevent damage of LLINs and properly care for them, or revise the current three-year LLIN distribution campaign schedule. Further research is needed to understand the determinants of physical integrity and attrition of LLINs.

## Data Availability

All the datasets are available on reasonable request to ACIPH.
